# Pulmonary arterial hypertension: the case for a bioelectronic treatment

**DOI:** 10.1186/s42234-019-0036-9

**Published:** 2019-12-10

**Authors:** Despοina Ntiloudi, Khaled Qanud, Jacquelyn-Nicole Tomaio, George Giannakoulas, Yousef Al-Abed, Stavros Zanos

**Affiliations:** 1 Institute of Bioelectronic Medicine, Feinstein Institutes for Medical Research, Manhasset, NY 11030 USA; 2 Department of Cardiology, AHEPA University Hospital, Thessaloniki, Greece

**Keywords:** Pulmonary arterial hypertension, Pathophysiology, Therapy, Bioelectronic medicine, Vagus nerve stimulation, Inflammation, Hemodynamics

## Abstract

Pulmonary arterial hypertension (PAH) is a rare disease of unknown etiology that progresses to right ventricular failure. It has a complex pathophysiology, which involves an imbalance between vasoconstrictive and vasodilative processes in the pulmonary circulation, pulmonary vasoconstriction, vascular and right ventricular remodeling, systemic inflammation, and autonomic imbalance, with a reduced parasympathetic and increased sympathetic tone. Current pharmacological treatments for PAH include several classes of drugs that target signaling pathways in vascular biology and cardiovascular physiology, but they can have severe unwanted effects and they do not typically stop the progression of the disease. Pulmonary artery denervation has been tested clinically as a method to suppress sympathetic overactivation, however it is a nonspecific and irreversible intervention. Bioelectronic medicine, in particular vagus nerve stimulation (VNS), has been used in cardiovascular disorders like arrhythmias, heart failure and arterial hypertension and could, in principle, be tested as a treatment in PAH. VNS can produce pulmonary vasodilation and renormalize right ventricular function, via activation of pulmonary and cardiac vagal fibers. It can suppress systemic inflammation, via activation of fibers that innervate the spleen. Finally, VNS can gradually restore the balance between parasympathetic and sympathetic tone by regulating autonomic reflexes. Preclinical studies support the feasibility of using VNS in PAH. However, there are challenges with such an approach, arising from the need to affect a relatively small number of relevant vagal fibers, and the potential for unwanted cardiac and noncardiac effects of VNS in this sensitive patient population.

## Background

Pulmonary arterial hypertension (PAH) is a rare but severe disease. The estimated prevalence rate is between 6.6-26.0 cases per million, and the estimated incidence rate is between 1.1-7.6 cases per million adult inhabitants per year (Badesch et al. 2010; Escribano-Subias et al. 2012; Humbert et al. 2006; Ling et al. 2012; Peacock et al. 2007). The mean age of the patients, in the past, was 36 years, while today the mean age at diagnosis is between 50 and 65 years (Badesch et al. 2010; Foley et al. 2011; Hoeper et al., 2013b; Rich et al. 1987). In developing countries, the baseline data of patients with PAH remain largely unchanged (Jing et al. 2007).

Over the last decades, survival rates of PAH patients have increased significantly (Thenappan et al. 2007). The main contributors to this outcome are earlier diagnosis due to increased awareness of the disease, referral to expert centers, administration of specific PAH therapy and improved special support strategies (Benza et al. 2012). However, the prognosis remains dismal: according to the REVEAL registry, the median survival of PAH patients is about 7 years (Benza et al. 2012). The progressive, fatal nature of the disease, combined with the high cost of pharmacotherapy and hospitalizations, has led to extensive research efforts focused on the development of new treatment options (Anand et al. 2016; McLaughlin et al. 2009). The scope of this article is to review the pathophysiological pathways of PAH, to highlight the existing treatment options and their limitations, and to discuss the potential therapeutic use of a bioelectronic therapy based on vagus nerve stimulation.

### Pathophysiology of PAH

In general, pulmonary circulation is a low pressure, low resistance system. PAH is defined as mean pulmonary arterial pressure (PAP) ≥25 mmHg (in the proposed new definition the cut off for mean PAP is even lower (>20mmHg)) with pulmonary arterial wedge pressure ≤15mmHg and pulmonary vascular resistance ≥3 Wood units (Galie et al. 2016; Simonneau et al. 2019). Arterial abnormalities in PAH cause the increase of pulmonary vascular resistance, which leads to a restriction of blood flow (Farber and Loscalzo 2004). The right ventricle (RV) becomes initially hypertrophic as a consequence of the increased afterload, which ultimately leads to right heart failure. The main mechanisms that cause the arterial abnormalities include vasoconstriction, endothelial-cell and smooth-muscle cell proliferation, in situ thrombosis, inflammation and formation of plexiform lesions (Archer and Rich 2000).

Chronic autonomic imbalance is common in PAH (Vaillancourt et al. 2017), with increased sympathetic (Nootens et al. 1995; Velez-Roa et al. 2004) and decreased parasympathetic activity (da Silva Goncalves Bos et al. 2018; Hemnes and Brittain 2018). In addition to, or perhaps partly because of, autonomic imbalance, the balance between vasodilators and vasoconstrictors, is disturbed in PAH (Farber and Loscalzo 2004). The production of vasodilators such as prostacyclin and nitric oxide (NO) are decreased in contrast with the production of vasoconstrictors, including endothelin and thromboxane, which are increased (Christman et al. 1992; Giaid et al. 1993). Furthermore, increased plasma levels of serotonin (5-hydroxytryptamine) might also play a role as vasoconstrictor and promoter of pulmonary artery smooth muscle cell proliferation in PAH (Herve et al. 1995; McLaughlin et al. 2009). Overexpression of 5-hydroxytryptamine transporter was associated with the latter outcome (Eddahibi et al. 2001; Marcos et al. 2004). Dysfunction of voltage-gated K+ channels lead also to pulmonary artery smooth muscle cell proliferation as well as vasoconstriction (Yuan et al. 1998).

Inflammation likely plays an important role in pathogenesis and progression of PAH , as well as in the development of RV failure (Kherbeck et al. 2013; Price et al. 2012; Rabinovitch et al. 2014; Voelkel et al. 2016). In patients with PAH, there is histologic evidence of accumulation of inflammatory cells and elevated levels of cytokines and chemokines (Huertas et al. 2014; Humbert et al. 2019), both around pulmonary vessels (Nicolls and Voelkel 2017) as well as in the failing RV (Sun et al. 2017). The elevated cytokine levels are associated with impaired RV function (Prins et al. 2018) and reduced 5-year survival (Soon et al. 2010). Another evidence of immune dysregulation is the lymphoid neogenesis in the lungs of idiopathic PAH patients (Perros et al. 2012). Lymphoid neogenesis is related to chronic inflammatory processes, such as autoimmunity and infection (Aloisi and Pujol-Borrell 2006).

Other pathophysiologic pathways that are involved in PAH development are the imbalance in antithrombotic/ prothrombotic factors and growth inhibitors/ mitogen factors (Farber and Loscalzo 2004). Decreased apoptosis can be evoked due to mutations in the transforming growth factor-beta receptor pathway, such as in the bone morphogenetic protein receptor 2 and in the activin receptor-like kinase 1 and endoglin, which are associated with familial PAH (Lane et al. 2000; Trembath et al. 2001). Furthermore, the renin-angiotensin-aldosterone (RAA) system is up-regulated and in specific angiotensin II type 1 receptors expression and signaling which is increased in pulmonary arteries of patients with idiopathic PAH, is associated with pulmonary artery smooth muscle cell proliferation (de Man et al., 2012b).

### Current therapies of PAH

#### Pharmacologic therapies

##### Calcium-channel blockers

Approximately 10% of PAH patients have a positive acute vasoreactivity test (Galie et al. 2016). This is defined as a fall in mean pulmonary artery pressure (mean PAP) of ≥10 mmHg to ≤40 mm Hg, with an unchanged or increased cardiac output after the administration of inhaled NO or iloprost, or intravenous epoprostenol or adenosine. Those patients are treated with high dose, progressively titrated, calcium channel blockers (Galie et al. 2016; Rich et al. 1992). However, if the patient after treatment with high dose of calcium channel blockers is in World Health organization (WHO) functional class III/ IV or his hemodynamic profile does not show marked improvement, initiation of specific PAH therapy is recommended (Galie et al. 2016). A study of Sitbon et al. showed that almost half of the acutely-vasoreactive patients were not long-term responders (Sitbon et al. 2005). Side effects of calcium channel blockers include hypotension, syncope and acute RV failure (Galie et al. 2016).

For all other cases, specific PAH therapy is followed and has three main pathophysiologic targets: endothelin, NO/cyclic guanosine monophosphate (cGMP), and prostacyclin.

### Endothelin pathway

Bosentan, ambrisentan and macitentan are endothelin receptor antagonists. They are beneficial in PAH patients, improving their exercise capacity, WHO functional class and their hemodynamics, while slowing disease progression (Channick et al. 2001; Galie et al. 2008a, 2008b; Pulido et al. 2013; Rubin et al. 2002) The main adverse effects of endothelin receptor antagonists are abnormal liver function, increased incidence of peripheral edema and anemia (McLaughlin et al. 2009).

### NO/cGMP pathway

Two types of drugs belong to the NO/cGMP pathway: phosphodiesterase type 5 inhibitors (sildenafil and tadalafil), which inhibit the degradation of cGMP, and riociguat, a guanylic cyclase stimulator that enhances cGMP production (Schermuly et al. 2008). Phosphodiesterase type 5 inhibitors were shown to improve the exercise capacity, WHO functional class, hemodynamics, quality of life, disease progression (Galie et al. 2009; Galie et al. 2005; Ghofrani et al. 2013; Sastry et al. 2004; Singh et al. 2006) and reduced N-terminal pro B-type natriuretic peptide (Ghofrani et al. 2013). The most common adverse events were flushing, diarrhea and dyspepsia for sildenafil and headache and myalgia and flushing for tadalafil (Galie et al. 2009; Galie et al. 2005; Sastry et al. 2004). Syncope is the most common serious adverse events of riociguat (Ghofrani et al. 2013).

### Prostacyclin pathway

Epoprostenol, iloprost, beraprost, treprostinil and selexipag constitute the category of drugs targeting the prostacyclin pathway. Epoprostenol is administered continually intravenously, iloprost requires multiple inhalations, usually six to nine times per day, treprostinil is administered subcutaneously, intravenously, inhaled and orally, while beraprost and selexipag is an orally administered selective prostacyclin receptor agonist. They have been shown to improve PAH symptoms, hemodynamics and exercise capacity and delay clinical worsening, while epoprostenol is the only compound to demonstrate reduced mortality in PAH patients (Badesch et al. 2000; Barst et al. 1996; Galie et al. 2002; Olschewski et al. 2002; Rubin et al. 1990; Simonneau et al. 2002; Sitbon et al. 2015). Common adverse events of prostanoids are headache, flushing, nausea, diarrhea, skin rash, musculoskeletal pain, jaw pain, infusion site pain for subcutaneous administration of treprostinil, and line infections for intravenous administration of epoprostenol (McLaughlin et al. 2009).

Regarding inhaled vasoactive intestinal peptide (VIP) and tyrosine kinase inhibitors, even though they theoretically seem promising medical therapies for PAH (Ghofrani et al. 2005; Leuchte et al. 2008; Nayyar et al. 2014; Petkov et al. 2003), their role in PAH treatment is controversial due to the recent negative clinical studies(Frost et al. 2015; Hoeper et al. 2013a,). Administration of imatinib although resulted in improved hemodynamics and exercise capacity, it increased significant adverse events and showed high discontinuation rate (Frost et al. 2015). The results from a randomized multicenter clinical trial phase II with subcutaneously administered VIP analogue are anticipated (NCT03556020).

Given that the PAH-specific therapy targets three different signaling pathways, in the case of inadequate treatment response or clinical worsening it is recommended that therapy is increased with sequential combination (Galie et al. 2016). Upfront combination therapy in WHO functional class II or III treatment-naive patients has also improved clinical outcome and is increasingly used in the therapeutic strategy in PAH (Galie et al. 2015). The favorable clinical results of combination therapy are supported additionally by the results of recent meta-analyses (Fox et al. 2016; Lajoie et al. 2016). In this context, it is likely that new treatment modalities could be considered as part of combination therapeutic schemes, even in PAH-specific treatment-naive patients.

Referral for transplantation in PAH patients is now postponed due to the wide use of specific PAH therapy, but when the maximal combination therapy fails and the patients remain severely symptomatic, transplantation is the only therapeutic option (Galie et al. 2016) (de Perrot et al. 2012; Fadel et al. 2010; Taylor et al. 2006; Toyoda et al. 2008).

### Drug therapies targeting the autonomic nervous system

#### Alpha/Beta adrenergic receptor blockers

Several mechanisms of action of beta blockers might support their use in PH. At the cellular level, beta blockers decrease RV myocardial hypertrophy and fibrosis, capillary rarefaction, apoptosis and inflammation (Bogaard et al. 2010; de Man et al. 2012a, 2013; Ishikawa et al. 2009; Perros et al. 2015). With regards to RV function, beta blockers reduce RV hypertrophy, increase RV and LV ejection fraction, decrease myocardial oxygen consumption and RV afterload (Perros et al. 2017). However, due to the reduction of heart rate and cardiac output, as well as the negative impact on exercise capacity (Bandyopadhyay et al. 2015; Provencher et al. 2006; Thenappan et al. 2014; van Campen et al. 2016), current guidelines do not recommend their use in PH patients except for those with comorbidities such as arrhythmia, high blood pressure, coronary artery disease and left heart failure (Galie et al. 2016).

#### ACE inhibitors/Angiotensin-1 receptor inhibitors/ Mineralocorticoid receptor antagonists

Dysregulation of renin–angiotensin-aldosterone system contributes to the pathophysiology of PAH (de Man et al. 2012b; Morrell et al. 1995). At a cellular level, this activation promotes vasoconstriction, cell proliferation, migration, extracellular matrix remodeling and fibrosis (Maron and Leopold 2014). Small studies with angiotensin-converting-enzyme (ACE) inhibitors, such as captopril, had conflicting results. Some studies had positive results, lowering the mean PAP and increasing the RV ejection fraction (Alpert et al. 1992; Ikram et al. 1982), but in others captopril had no effect in pulmonary circuit (Leier et al. 1983; Rich et al. 1982). Studies with angiotensin-1 receptor inhibitors were also inconclusive. In animal models, losartan was reported to delay disease progression, decrease RV afterload and pulmonary vascular remodeling and restore RV-arterial coupling (de Man et al. 2012b); however, in other studies failed to prevent or reduce the ventricular afterload (Cassis et al. 1992; Kreutz et al. 1996). Only one small clinical study showed that losartan is not inferior to nifedipine with regards to mean PAP and exercise capacity (Bozbas et al. 2010). Since aldosterone plasma levels are high in PAH patients (Maron et al. 2013a) mineralocorticoid receptor antagonists were also tested as a possible therapeutic target (Maron et al. 2012; Preston et al. 2013). Indeed, in the trials for ambrisentan (ARIES) it was noted that patients treated with ambrisentan plus spironolactone had a trend toward better functional capacity and plasma-B-type natriuretic peptide (Maron et al. 2013a). However, there are not large clinical trials to support the role of these drugs in PAH patients, data from ongoing clinical trials are going to define their efficacy and torelability in these patients (Clinical-Trials.gov Identifier: NCT01712620, NCT03177603).

In general, the several classes of drugs targeting the autonomic nervous system and the renin-angiotensin-aldosterone system, albeit the positive preclinical findings, have not shown definite clinical benefit and not widely recommended in PAH (Table 1).

**Table 1:** Pharmacological and invasive therapies targeting the autonomic nervous system in pulmonary arterial hypertension

Therapy	Mechanism of action in PAH	Drawbacks	Recommendation
Alpha/ Beta blockers	Reduction in: - Right ventricular myocardial hypertrophy and fibrosis -capillary rarefaction -apoptosis -inflammation	Reduction in heart rate, cardiac output and exercise capacity	PH patients with comorbidities (arrhythmia, high blood pressure, coronary artery disease, left heart failure)
Renin-angiotensin-aldosterone system inhibitors	Reduction in: -vasoconstriction -cell proliferation -extracellular matrix remodeling -fibrosis	No beneficial effect in some studies	More clinical data needed
Sympathetic ganglion block	Suppression of activation of the sympathetic nervous system (SNS)	-Non-specific -Invasive -Not readily reversible -No clinical data	More data needed
Renal artery denervation	Suppression of activation of the SNS and renin-angiotensin-aldosterone system	-Non-specific -Invasive -Irreversible -No clinical data in PH	More data needed
Pulmonary artery denervation	Suppression of activation of the SNS	-Non-specific -Invasive -Irreversible	Being tested clinically

### Invasive therapies

Pulmonary artery denervation (PAD) is a clinically-tested non-pharmacological treatment of PAH. Given that sympathetic nerve activity in PAH is increased (Velez-Roa et al. 2004), ablation at the main bifurcation area of the left pulmonary artery (PAD) has been attempted as a treatment option in PAH (Chen et al. 2013a). In the preclinical context, PAD induced sympathetic nerve injury, including axon loss, demyelination, prolonged conduction time and loss of potential amplitude, improved the hemodynamics, reducing the mPAP and pulmonary vascular resistance and caused pulmonary artery remodeling (Chen et al. 2013b; Rothman et al. 2015; Zhou et al. 2015). In a single-center clinical study of PAD, favorable outcomes were reported with respect to hemodynamics, functional capacity and cardiac function assessed by echocardiography (Chen et al. 2013a; Chen et al. 2015). In a recent multicenter, randomized study, PAD improved hemodynamic and clinical outcomes compared to sildenafil in patients with combined pre- and post-capillary pulmonary hypertension associated with left heart failure (H. Zhang et al. 2019).

Even though the results of these non-controlled trials should be interpreted with caution, the effectiveness of PAD in PAH suggests that targeting the autonomic nervous system (ANS) in PAH via an invasive method could be a viable therapeutic strategy. Sympathetic ganglion block is an experimental therapy that, like PAD, also targets the over-activation of the sympathetic nervous system. Superior cervical ganglion or stellate ganglion block using local injections of an anesthetic agent have been tested in rodent models of PAH, with encouraging results (Na et al. 2014). Finally, catheter-based renal artery denervation, an intervention that targets the activation of the sympathetic nervous system and the RAA system, has been tested in a canine (Qingyan et al. 2015) and a rodent (Liu et al. 2017) model of PAH, again with encouraging results.

Drawbacks of these invasive approaches is that they are non-specific, since the ablation or block takes place without targeting specific branches or fiber types of the neural structures and that they are completely or partially irreversible (Table 1). This raises the possibility that a targeted, reversible method for modulating autonomic tone may be another viable, perhaps preferable, therapeutic approach in PAH.

### Bioelectronic medicine and its cardiovascular applications

#### Principles of bioelectronic medicine therapies

Placement of neurostimulation probes in contact with nerves of the ANS to deliver therapeutic neuromodulation in diseases or conditions in which the ANS is implicated is a relatively new family of interventions, for which the terms “electroceuticals”, “bioelectronic medicine” (BEM) (Famm et al. 2013) or “autonomic regulation therapies” (Dicarlo et al. 2013; Premchand et al. 2014) have been used. The basis of BEM therapies is that up- or down-modulation of the tone in specific parts of the ANS can have predictable effects on the afferent (sensory) information that is conveyed to the brain and on the efferent (motor) commands modulating the function of innervated organs. These effects can be acute or chronic, and typically involve alterations in the function of several organs and systems, depending on which nerve targets and fibers are stimulated, which autonomic reflexes are recruited by stimulation and which physiological adaptations occur in response to neurostimulation. BEM leverages these principles to develop neurostimulation therapies that target specific mechanisms and neural circuits that are affected or implicated in different diseases.

Targeting nerves with electrical stimulation to treat diseases has two major advantages over pharmaceutical therapies. First is specificity with regards to the targeted organ system: placing the probe on a specific nerve and using fiber-selective electrical stimulation waveforms can deliver the therapy specifically to the affected organ while engaging only the relevant autonomic fibers (Birmingham et al. 2014). Drugs, on the other hand, are delivered systemically and affect receptors on all tissues and organs. Second is specificity with regards to time: the timing of delivery of neurostimulation can be tightly controlled, even triggered by specific events or physiological states and not by others, i.e. only when therapy is needed and not continuously (Zanos 2018). Pharmaceuticals have typically sustained presence in the organism, determined by pharmacokinetics with time course that in not under our control once the drug is delivered. The cardiovascular system is controlled by many autonomic nerves and fibers and, in addition, has a highly dynamic physiology. For these reasons, cardiovascular diseases like PAH are, in principle, good candidates for BEM therapies.

The vagus nerve is a major autonomic nerve with increased therapeutic potential, for 2 reasons: first, because its afferent and efferent arms are involved in the sensory and motor innervation of practically all organs and, second, because its surgical approach at the cervical region is well-established, relatively easy, can be performed as an outpatient procedure.

### Bioelectronic medicine therapies of cardiovascular disorders

The heart and vessels are heavily innervated by the ANS, both its sympathetic and parasympathetic arms. The autonomic innervation is involved in the continuous physiological control of cardiovascular function (Armour 2004; Hanna et al. 2017) and is implicated in the acute and chronic adaptive and maladaptive pathophysiological responses to diseases affecting the cardiovascular system (Armour 2004). As a result, targeting the autonomic innervation of the cardiovascular system using neurostimulation has been one of the first applications of the BEM approach, aimed at disorders like hypertension, heart failure and cardiac arrhythmias (Horn et al. 2019). For example, electrical stimulation of the carotid sinus nerve via an implantable device reduces blood pressure in some patients with drug-resistant hypertension by modulating the tone of the baroreflex (de Leeuw et al. 2017; Scheffers et al. 2010). Device-based interventions that target the cardiac vagus or the cardiac sympathetic nerves have successfully suppressed or prevented atrial and ventricular arrhythmias, both in animal models and in clinical trials (Waldron et al. 2019; Zhu et al. 2019).

More specifically, the therapeutic effects of VNS in experimental models of heart failure is of relevance to its potential use in the context of PAH. Stimulation of the cervical vagus in animal models of heart failure reduced heart rate, improved the systolic and diastolic function of the left ventricle, reversed left ventricular hypertrophy, and reduced the frequency of arrhythmias and sudden cardiac death (Sabbah et al. 2011); favorable effects were also documented in a clinical trial (Premchand et al. 2014). Some of these effects were independent of the VNS-induced reduction in heart rate (Y. Zhang et al. 2009); instead, several neural mechanisms, at multiple levels of the cardiac autonomic nervous system, have been implicated in these actions of VNS, including activation of the baroreflex (Y. Zhang et al. 2009) and modulation of intrathoracic cardio-cardiac and central reflexes (Hanna et al. 2018). In addition, VNS may favorably affect cellular and structural markers of remodeling in the failing left ventricle (Beaumont et al. 2015; Sabbah et al. 2011). There is evidence that VNS might exert such actions through increased production of nitric oxide in the myocardium, down-regulation of gap junction proteins, changes in neural excitability in the intrinsic cardiac nervous system, in the metabolism of cardiomyocytes and apoptosis-related proteins (Sabbah et al. 2011).

### The rationale for a bioelectronic treatment of pulmonary hypertension

A BEM therapy of PAH, more specifically a therapy based on vagus nerve stimulation (VNS), would in principle target several major pathogenetic and pathophysiologic mechanisms, namely, pulmonary vasoconstriction, right ventricular dysfunction and systemic inflammation, as well as chronic autonomic imbalance (Fig. 2).

### Targeting pulmonary vasoconstriction and right ventricular dysfunction

**Fig. 1:**
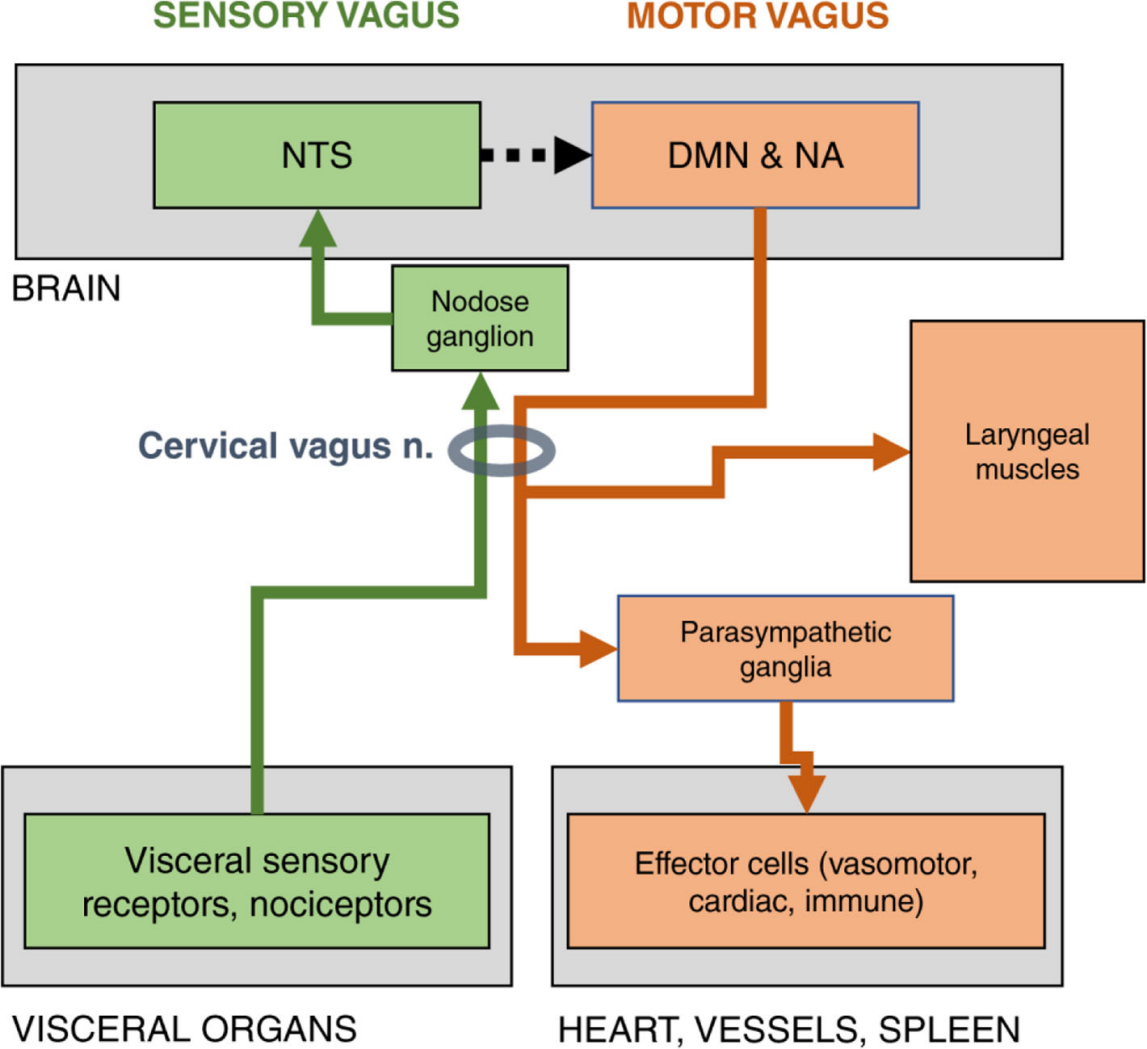
Schematic diagram of the main central and peripheral components of the motor and sensory vagus. The vagus nerve includes sensory (afferent) and motor (efferent) arms, both of which are represented in the cervical region, where vagus cuffs are typically implanted. The sensory vagal pathway, shown in green, originates with general sensory receptors (i.e. nociceptors) in visceral organs, including vessels, the lungs, the heart, the gastrointestinal tract, the liver, lymph nodes etc. They convey information about mechanical parameters, e.g. increased wall tension in vessels in high blood pressure or in lung alveoli during inhalation, or biochemical events, e.g. presence of bacteria or inflammatory cytokines at an injured site. Afferent fibers from these receptors synapse at sensory neurons in the nodose ganglion of the vagus, located at the height of the transverse process of the first cervical vertebra. Axons of those sensory ganglionic neurons project to the nucleus of the solitary tract (NTS), in the brainstem. The motor vagal pathway, shown in red, originates in the motor nuclei of the vagus in the brainstem, the dorsal motor nucleus (DMN) and the nucleus ambiguous (NA). Peripheral axons of those cells go through the cervical vagus, and either innervate laryngeal muscles, or synapse on neurons in parasympathetic ganglia, close to organs like the heart, the lungs, the intestine, the pancreas, etc. An important target of the motor vagus is the spleen; in this case, motor vagal fibers synapse at the celiac ganglion, from which adrenergic fibers project to the spleen

Vasoconstriction is one of the pathophysiological mechanisms of PAH. It is well-established that the autonomic nervous system is regulating pulmonary vascular tone (Farber and Loscalzo 2004; Hemnes and Brittain 2018; Mouratoglou et al. 2016). The respiratory track receives sympathetic innervation from neurons whose cell bodies reside mainly in the stellate ganglion and thoracic sympathetic chain ganglia T2-T5 (Kummer et al. 1992). The density along with the vascular reactivity to neurotransmitters decrease towards the periphery (Kummer 2011). The sympathetic nervous system causes vasoconstriction in lung vasculature and the responsible post-ganglionic neurotransmitter, norepinephrine, is reported to decrease compliance and increase resistance in pulmonary vascular bed (Knight et al. 1981). Both decreased compliance and increased resistance play a role in the development of PAH (Saouti et al. 2010). Intrapulmonary vessels are also innervated by parasympathetic neurons, which originate mainly from nucleus ambiguus (Hadziefendic and Haxhiu 1999) and provide cholinergic innervation through bronchopulmonary vagal branches (Fig. 1). Similarly to the sympathetic, the density of the parasympathetic fibers in the lung decreases towards the periphery; in addition, parasympathetic innervation is sparser compared to sympathetic (Kummer 2011). Stimulation of the efferent vagal nerve releases acetylcholine, which through a nitric oxide-dependent mechanism, causes dilation to the pulmonary vascular bed (McMahon et al. 1992). Acetylcholine can also downregulate the release of norepinephrine, acting on adrenergic terminals (Knight et al. 1981).

VNS could exert its protective effects by altering the function of the right ventricle (RV). VNS has been shown to increase RV contraction and relaxation (Henning et al. 1996). In a preliminary study in a rodent model of RV pressure overload, a condition that mimics RV dysfunction and the progressive development of RV failure in the presence of pulmonary hypertension, VNS normalized several indices of RV systolic function (Yoshida et al. 2018a). Some of those indices, for example RV end-systolic elastance, are independent of RV afterload, suggesting that VNS may be directly benefiting RV compensation to increased pressure in the pulmonary artery.

Along with pulmonary vasodilation and a direct effect on RV function, another potential, hemodynamically beneficial effect of VNS is suppression of supraventricular arrhythmias, with are most common in PAH patients (Y. Zhang and Mazgalev 2011; Zhu et al. 2019). Such a protective effect of VNS has been documented in post-operative patients receiving low-level VNS (Stavrakis et al. 2017) and, recently, in a population of patients newly diagnosed with atrial fibrillation (Stavrakis et al. 2019).

### Targeting systemic inflammation

One of the well-established effects of VNS is down-modulation of the immune response to an acute inflammatory challenge, like the injection of lipopolysaccharide, a response that initiates in the spleen and includes coordinated production of inflammatory cytokines (Pavlov et al. 2018). A series of studies established a neuro-immune reflex, the inflammatory reflex, with an afferent and an efferent arm (Fig. 1). Briefly, the afferent arm starts with the axons of afferent neurons innervating the viscera; these neurons respond to noxious stimuli, fragments of pathogens and cytokines released by immune cells. Vagal sensory neurons have cell bodies in the nodose ganglion and project to the nucleus of the solitary tract in the brainstem. The efferent arm starts in the dorsal motor nucleus of the vagus, in the brainstem, continues with the efferent vagal fibers and synapses in the celiac ganglion; from there, noradrenergic neurons project to the spleen, where release of norepinephrine activates the release of choline acetyl-transferase (ChAT) positive CD4+ T-cells. In turn, these ChAT cells release acetyl choline, which acts on macrophages and other immune cells through α7 nicotinic receptors, resulting in suppression of proinflammatory cytokine production.

Since production and release of cytokines are central in the development of the acute and chronic inflammatory response, the physiologic and pathophysiologic role of the vagus in inflammation has been studied extensively (Pavlov et al. 2018). Activating the vagus system via VNS results in suppression of the release of tumor necrosis factor, interleukin1beta and other cytokines, and amelioration of the clinical and pathological consequences of inflammation. This has been successfully tested in animal models of endotoxemia (Borovikova et al. 2000), hemorrhagic shock (Guarini et al. 2004), sepsis (Huston et al. 2006), arthritis (Levine et al. 2014) and other autoimmune diseases (Tracey 2007), heart failure (Y. Zhang et al. 2009) etc. Suppression of the inflammatory reflex using VNS is explored as a therapeutic modality in clinical trials in patients with rheumatoid arthritis (Genovese et al. 2019; Koopman et al. 2016), lupus (Aranow et al. 2018) and inflammatory bowel disease (Bonaz et al. 2016).

In the context of PAH, suppression of systemic inflammation by VNS could ameliorate the pathological inflammatory process in the pulmonary vessels, slowing down or reversing vascular remodeling (Nicolls and Voelkel 2017), and in the right ventricle, protecting it from hypertrophy and maladaptive remodeling (Sun et al. 2017).

### Targeting autonomic imbalance

**Fig. 2:**
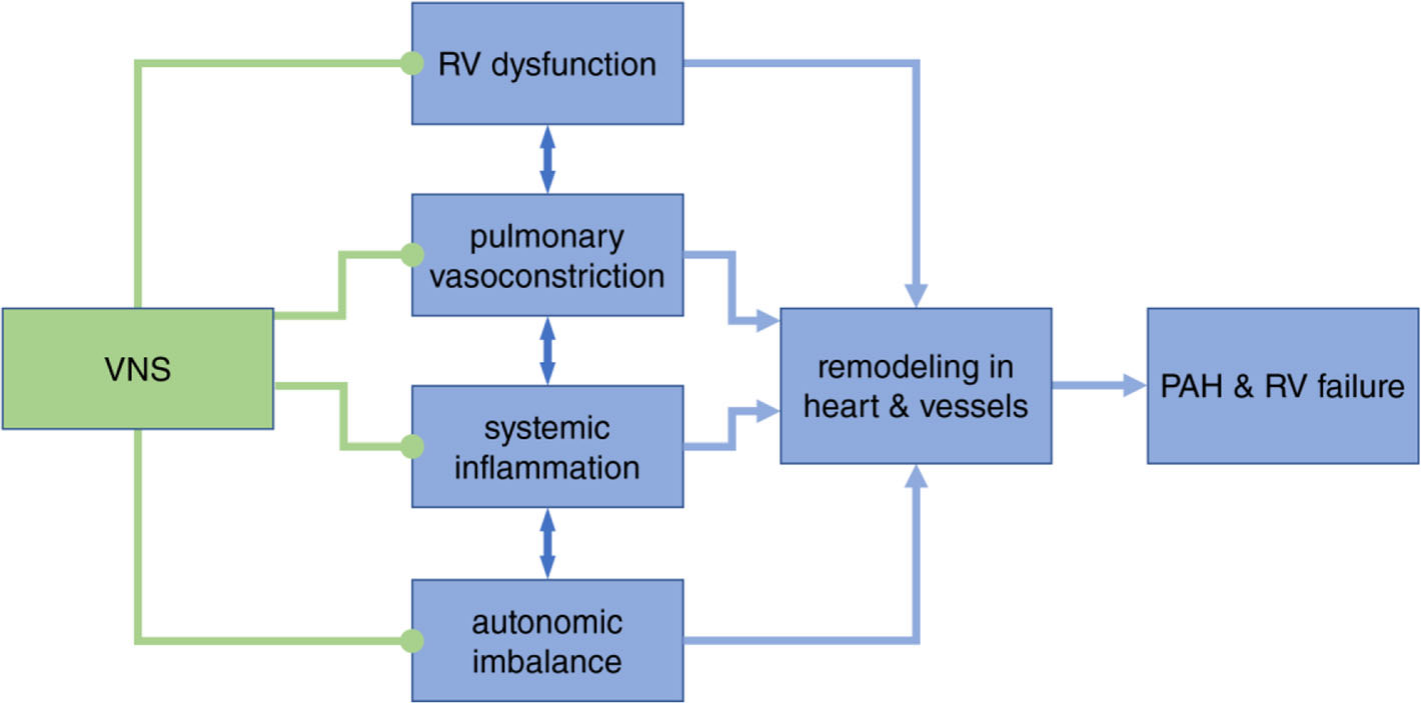
Potential actions of VNS on different pathogenetic mechanisms involved in PAH. Pulmonary arterial vasoconstriction, right ventricular (RV) dysfunction and systemic inflammation are core mechanisms in the pathogenesis of PAH. They may be related to autonomic imbalance that is common in PAH, with an increased sympathetic and decreased parasympathetic tone. Vasoconstriction, RV dysfunction and inflammation lead to remodeling in the RV and pulmonary vessels, which eventually exacerbate the pathophysiology of PAH. In principle, VNS could target therapeutically these mechanisms in the following ways: VNS produces NO-mediated vasodilation in the pulmonary circulation via vagal bronchopulmonary branches. VNS renormalizes RV function via efferent and possibly afferent cardiac vagal fibers. VNS down-modulates cytokine-mediated immune response via branches that terminate in the spleen. VNS chronically restores autonomic imbalance, possibly via re-setting vagal and non-vagal autonomic reflexes between the brain and periphery

In addition to the “direct” actions of VNS on pulmonary vasoconstriction and systemic inflammation, chronic VNS may gradually improve the imbalance between the sympathetic and parasympathetic tone which may contribute to, or be caused by, the inflammatory and hemodynamic aspects of PAH pathogenesis (Ameri et al. 2016) (Fig. 2). In a recent study in a rodent model of PAH, it was shown that VNS for several weeks attenuated pulmonary vascular remodeling, preserved RV function and improved survival (Yoshida et al. 2018b); it accomplished that by chronically “re-setting” the balance between sympathetic and parasympathetic reflex circuits in the periphery and in the central nervous system (Kingma et al. 2018).

### Challenges for a bioelectronic treatment of pulmonary hypertension

The direct, motor component of vasodilatory and anti-inflammatory effects of VNS is primarily mediated by preganglionic, efferent, B-type, cholinergic vagal fibers. Therefore, a VNS-based treatment of PAH shouldprimarily target cholinergic B-type fibers, ideally those innervating the pulmonary vessels and the spleen. Such an approach would have to overcome several challenges.

First, large, myelinated efferent A-type vagal fibers, some of which innervate muscles of the larynx and pharynx, have lower activation thresholds that the efferent, parasympathetic B-type fibers. Activation of those larger fibers gives rise to adverse effects like coughing, voice hoarseness, nausea etc., and frequently limits the intensity of cervical VNS to levels that are sub-therapeutic with regards to B-fibers. A potential solution could be to deliver stimulation to the cervical vagus that spares A-type and only targets B-type fibers using appropriate electrodes and stimulation waveforms, an area of active preclinical investigation (Guiraud et al. 2016; Musselman et al. 2019; Patel and Butera 2018).

Second, activation of large, myelinated afferent A-type fibers by VNS, again with lower activation thresholds than B-type fiber, may induce a reflexive decrease in parasympathetic tone and increase in sympathetic tone (Ardell et al. 2017). In addition, there is a small number of B-type, sympathetic, catecholaminergic fibers in the human vagus (Seki et al. 2014), and their activation by VNS could in principle directly enhance the sympathetic tone to the heart and vessels or stimulate sympathetic reflexes. Whatever the mechanism, a VNS-induced increase in sympathetic tone might exacerbate the pathophysiology of PAH. Therefore, it is important for a cervical VNS therapy to exert precise control over the relative amounts of the afferent and efferent parasympathetic and sympathetic activation, both direct and reflexive. Such cardiovascular control has been demonstrated experimentally in the cardiac vagus by fine-tuning specific VNS parameters, pulse width, intensity and pulsing frequency, according to the “neural fulcrum” hypothesis (Ardell et al. 2017).

Third, even if preganglionic, cholinergic fibers are successfully targeted, many of them innervate the heart. Cholinergic B-type fiber-specific VNS at the cervical level could have negative chronotropic, dromotropic and inotropic cardiac effects (Coote 2013), compromising the hemodynamic condition of patients with PAH who are sensitive to drops in cardiac output. A potential solution could be to use stimulation probes that specifically target fibers that innervate the lungs and/or the spleen. That could be attained, in principle, by implanting the probes closer to the end-organs, rather than at the cervical level. However, that would require more invasive surgery, as implanting a stimulation probe at the bronchial branch, or branches, of the vagus would require a thoracotomy, whereas implanting it at the splenic nerve would require a laparotomy. Thoracoscopy or laparoscopy could in principle be used instead. Alternatively, a cervical vagus electrode that specifically targets the pulmonary or splenic fibers could be used. That would require detailed knowledge of the branching pattern and radial distribution of pulmonary and splenic fibers at the level of the cervical vagus (Hammer et al. 2015), and fabrication of multi-contact, high-resolution stimulating electrodes that could target this kind of anatomical organization (Plachta et al. 2014).

Finally, activation of C-type afferent fibers by VNS might produce respiratory abnormalities (Coleridge and Coleridge 1984) including alterations in the breathing rhythm, bronchial mucus secretion, bronchoconstriction and cough (Undem and Kollarik 2005), and possibly changes in local bronchial and pulmonary neuroimmune and inflammatory reflexes, with unknown functional and clinical significance (Adriaensen and Timmermans 2011). However, activation of C-type fibers happens at much higher current intensities than those of B-type fibers (Heinbecker 1930) and can easily be avoided by calibration of VNS intensity (McAllen et al. 2018).

## Conclusions

PAH is a lethal disease of the pulmonary circulation and the right heart. Its complex pathophysiology involves, among others, chronic autonomic imbalance, in particular reduction of the parasympathetic tone, pulmonary vasoconstriction, chronic inflammation, and vascular remodeling. A bioelectronic medicine therapy, by stimulating the vagus nerve, can target several of these processes, as VNS produces pulmonary vasodilation, suppresses inflammation and restores autonomic balance. Preliminary studies in preclinical animal models of PAH point to potentially therapeutic effects of VNS and warrant further investigations. Physiological studies of the hemodynamic and anti-inflammatory effects of VNS in conditions and models that mimic PAH will help us understand the therapeutic potential of such an approach and drive patient selection criteria. Neurophysiological and biophysical studies in fiber-selective neurostimulation will result in modes of stimulation that recruit fibers in a desired manner, avoiding unwanted effects. Finally, anatomical and neural tracing studies of the organization of the vagal innervation of the lung vessels and the spleen in small and, primarily, in large animal models will guide the fabrication of more effective and selective stimulation probes for humans.

## Data Availability

Not applicable

## Abbreviations

ANS: Autonomic nervous system
BEM: Bioelectronic medicine
ChAT: Choline acetyl-transferase
PAP: Pulmonary artery pressure
NO: Nitric oxide
PAD: Pulmonary artery denervation
PAH: Pulmonary arterial hypertension
RV: Right ventricle
VNS: Vagus nerve stimulation
WHO: World Health Organization
